# Ubiquitin-Conjugating Enzyme Positively Regulates Salicylic Acid and Jasmonic Acid Biosynthesis to Confer Broad-Spectrum Antiviral Resistance in *Nicotiana benthamiana*

**DOI:** 10.3390/plants14203234

**Published:** 2025-10-21

**Authors:** Xianglong Zhang, Zihao Chen, Shijie Jiang, Lin Xie, Jingjing Fan, Nengbing Hu, Xiangxiang Zhang

**Affiliations:** Key Laboratory of Crop Germplasm Innovation and Green Production, Anhui Science and Technology University, Chuzhou 239000, China; m15215507542@163.com (X.Z.); zihaochen477@gmail.com (Z.C.); 17305656426@163.com (S.J.); xl183259@163.com (L.X.); 19556956368@163.com (J.F.); hunengbing@126.com (N.H.)

**Keywords:** UBC, turnip mosaic virus resistance, salicylic acid, jasmonic acid, VIGS

## Abstract

Ubiquitin-conjugating enzyme (UBC) plays a significant role in plant hormone signal transduction. In this study, we observed that TuMV infection markedly upregulates UBC mRNA expression, suggesting a close association between UBC and viral infection. Using Tobacco rattle virus (TRV)-based virus-induced gene silencing (VIGS) to downregulate UBC expression in *Nicotiana benthamiana*, we found that UBC-silenced plants exhibited enhanced susceptibility to TuMV compared with control plants. Conversely, transient overexpression of UBC protein suppressed viral propagation. Further analysis by reverse transcription quantitative PCR (RT-qPCR) revealed a substantial downregulation in the expression of SA and JA biosynthetic genes in UBC-silenced plants. Accordingly, liquid chromatography–tandem mass spectrometry (LC-MS/MS) confirmed a marked decrease in the accumulation of the corresponding hormones. Exogenous application of SA or JA partially restored antiviral resistance in UBC-silenced plants, indicating that hormonal deficiency contributes to enhanced viral susceptibility. Collectively, our results demonstrate that UBC positively regulates SA and JA biosynthesis. UBC silencing impairs both SA- and JA-mediated defense pathways, thereby facilitating viral infection in *N. benthamiana*.

## 1. Introduction

Turnip mosaic virus (TuMV) is a widely distributed single-stranded RNA virus [1]. TuMV infection causes symptoms such as leaf yellowing, deformation, and plant dwarfing, severely affecting crop yield and quality [2,3]. Plant hormones play important roles in defending against viral infection, with enhanced hormone signaling contributing to the regulation of plant immunity [4]. Jasmonic acid (JA) regulates plant immunity and enhances defense responses against fungi and insects [5]. The JA signaling pathway is mediated by the receptor protein CORONATINE INSENSITIVE 1 (COI1), which promotes the degradation of JAZ proteins, thereby releasing the activity of downstream transcription factors such as MYC2, MYB21, and ORA59; this subsequently induces the expression of resistance-related genes and enhances plant disease resistance [6]. Viruses can interfere with plant hormone signaling pathways to weaken host defense mechanisms. For example, the NIb protein encoded by TuMV targets the plant protein NPR1, blocking its interaction with SUMO3 and subsequent SUMOylation, thereby suppressing salicylic acid (SA)-mediated plant immunity [7]. The 2b protein of Cucumber mosaic virus (CMV) interferes with the interaction between COI1 and JAZ proteins, inhibiting JAZ degradation and thus blocking JA signaling transduction, which facilitates viral infection [8]. In addition, the βC1 protein of the Tomato yellow leaf curl China virus (TYLCCNV) interacts with MYC2 to disrupt its dimerization, thereby suppressing JA signaling and promoting viral infection [9]. The coat protein (CP) of the Citrus yellow vein clearing virus (CYVCV) interacts with the citrus protein ClAPX1 and enhances its enzymatic activity, reducing ROS and JA accumulation and suppressing plant defense pathways to facilitate viral infection [10]. Moreover, multiple viral proteins target the RAV-type transcription factor OsRAV15 to disrupt its interaction with OsMYC2, thereby inhibiting JA signaling transduction, decreasing JA accumulation, weakening broad-spectrum resistance to rice viruses, and promoting viral infection [11]. Oligosaccharin treatment in rice induces JA accumulation and reactive oxygen species (ROS) burst, significantly enhancing resistance to Rice black-streaked dwarf virus (RBSDV) [12].

Salicylic acid (SA) plays a crucial role in plant immune responses during viral infection. SA treatment in tomato mediates the upregulation of genes such as ascorbate peroxidase (APX) and peroxidase (POD) in the ROS pathway, thereby inducing the expression of pathogenesis-related genes and enhancing resistance to Tomato yellow leaf curl virus (TYLCV), suppressing viral infection [13]. The ultrahigh activity plant immune inducer (ZNC) enhances tobacco resistance to Potato virus X (PVX) by modulating both the salicylic acid (SA) pathway and the RNA silencing pathway [14]. However, viruses can also interfere with plant hormone signaling transduction, thereby weakening plant immunity. For instance, the 2b protein encoded by Cucumber mosaic virus (CMV) disrupts the RNA silencing pathway and significantly suppresses SA-mediated antiviral defense, thus facilitating viral infection [15].

E3 ubiquitin ligases play significant roles in plant hormone biosynthesis and signaling transduction. By recognizing specific substrates and mediating their ubiquitination, E3 ubiquitin ligases regulate the degradation or functional modification of target proteins [16]. In plants, E3 ubiquitin ligases are widely involved in hormone signaling pathways, cell cycle regulation, and stress responses [17,18,19]. Studies have shown that E3 ubiquitin ligases regulate the stability of key biosynthetic enzymes in hormone signaling pathways, such as 13-lipoxygenase (13-LOX) in the jasmonic acid (JA) pathway and phenylalanine ammonia-lyase (PAL) and isochorismate synthase 1 (ICS1) in the salicylic acid (SA) pathway, thereby modulating hormone accumulation [20,21,22]. In addition, E3 ubiquitin ligases regulate hormone signal transduction by ubiquitinating and modifying key transcription factors involved in plant defense responses [23]. JA-Ile induces the SCF^COI1 E3 ubiquitin ligase to bind and ubiquitinate the JAZ1 repressor, leading to its 26S-proteasome-mediated degradation and consequent release of MYC2 to activate jasmonate signaling [24]. Rice stripe virus (RSV) infection induces the upregulation of the OsGSK2 protein, which mediates the degradation of OsMYC2 through phosphorylation modification, thereby suppressing JA-activated immunity and promoting viral infection [25].

This study found that TuMV-GFP infection induces the upregulation of UBC mRNA expression, an E3 ubiquitin ligase. TuMV-GFP infection was significantly promoted in UBC-silenced plants, whereas transient expression of UBC inhibits viral infection. Furthermore, the mRNA-related gene expression of the JA and SA pathways was found to be significantly downregulated in UBC-silenced plants. Exogenous application of these hormones could partially restore plant resistance to the virus. In addition, UBC was shown to confer broad-spectrum resistance against viral infection. In conclusion, this study lays the foundation for understanding the mechanism by which E3 ubiquitin ligases participate in plant defense against viral pathogens.

## 2. Results

### 2.1. TuMV-GFP Infection Enhanced UBC mRNA Expression

**Figure 1:**
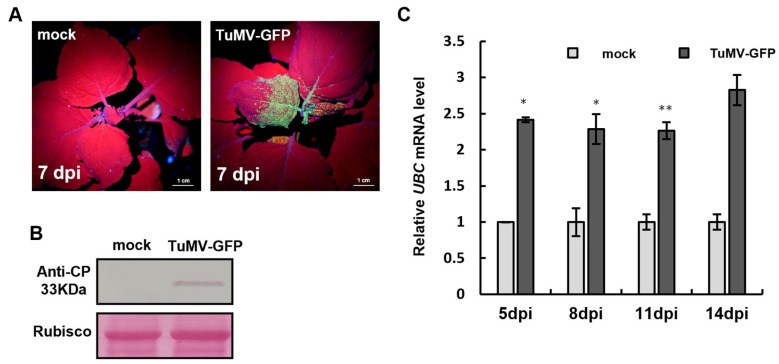
TuMV-GFP infection enhances UBC expression in *N. benthamiana*. Expression levels of ubiquitin-conjugating enzyme (UBC) transcripts in TuMV-GFP-infected *Nicotiana benthamiana*. (**A**) Systemic GFP fluorescence (indicative of TuMV-GFP infection) under UV light at 7 dpi in *N. benthamiana* (right) and a mock-inoculated plant (left). (**B**) TuMV-GFP CP detected by Western blot using anti-CP antibody at 7 dpi. Rubisco served as a loading control. (**C**) After TuMV-GFP infection, the level of expression of UBC transcripts was examined by RT-qPCR at 5 dpi, 8 dpi, 11 dpi, and 14 dpi. Experiments were repeated three times. Bars represent the standard errors of the means. A two-sample unequal variance directional *t*-test was used to test the significance of the difference (** *p* < 0.01, * *p* < 0.05).

TuMV-based viral vector encoding GFP (TuMV-GFP) was delivered into *N. benthamiana* leaves using Agrobacterium tumefaciens and Agrobacterium-mediated infiltration onto *N. benthamiana* plants, and at 7 days post-inoculation (dpi) (post-TuMV-GFP inoculation), the viral infection status was observed under UV illumination. Under UV light, the systemic leaves of TuMV-GFP-infected *N. benthamiana* exhibited high-green-fluorescence symptoms, whereas no green fluorescence was observed in the control plants (Figure 1A). Using antibodies specific to the TuMV coat protein (CP), Western blot analysis detected a distinct CP band in TuMV-GFP-infected *N. benthamiana*, while no CP band was detected in the control group, confirming successful TuMV-GFP infection (Figure 1B). To determine whether TuMV-GFP infection affects UBC mRNA expression, RT-qPCR was performed to measure UBC mRNA levels. Time points selected for detection included 5 dpi, 7 dpi, 11 dpi, and 14 dpi after TuMV-GFP infection. The results showed that UBC mRNA expression levels were higher in TuMV-GFP-infected plants (Figure 1C).

### 2.2. UBC Silencing Promoted TuMV-GFP Infection in N. benthamiana

**Figure 2:**
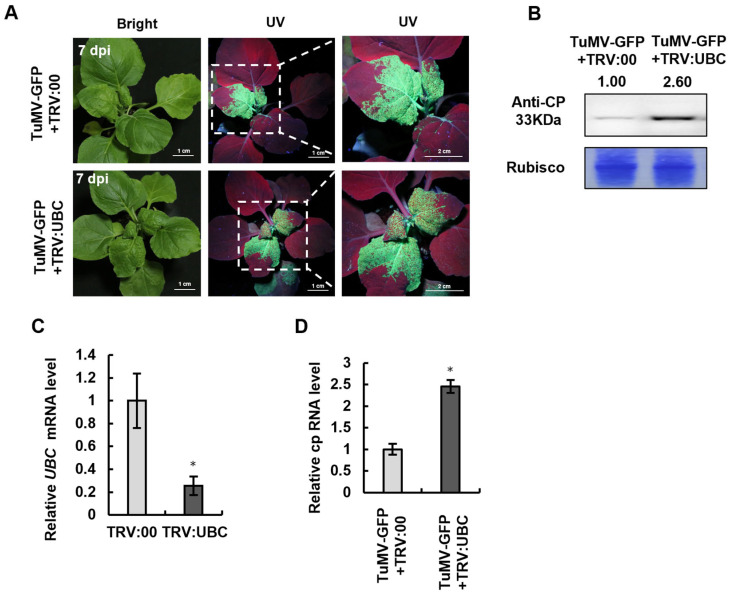
UBC silencing promoted TuMV-GFP infection in *N. benthamiana*. Increased TuMV-GFP accumulation in UBC-silenced *Nicotiana benthamiana*. (**A**) TuMV-GFP systemic infection in UBC-silenced or mock-inoculated *N. benthamiana* under UV. (**B**) Western blot of TuMV CP in TRV:UBC and TRV:00 plants at 7 dpi. Rubisco served as a loading control; band intensities were quantified with ImageJ and normalized to Rubisco. (**C**) After TRV-mediated UBC silencing, the accumulation of UBC was examined by RT-qPCR at 14 dpi. Experiments were repeated three times. (**D**) After TuMV-GFP infection, the accumulation of CP was examined by RT-qPCR at 7 dpi. Experiments were repeated three times. Bars represent the standard errors of the means. A two-sample unequal variance directional *t*-test was used to test the significance of the difference (* *p* < 0.05).

**Figure 4:**
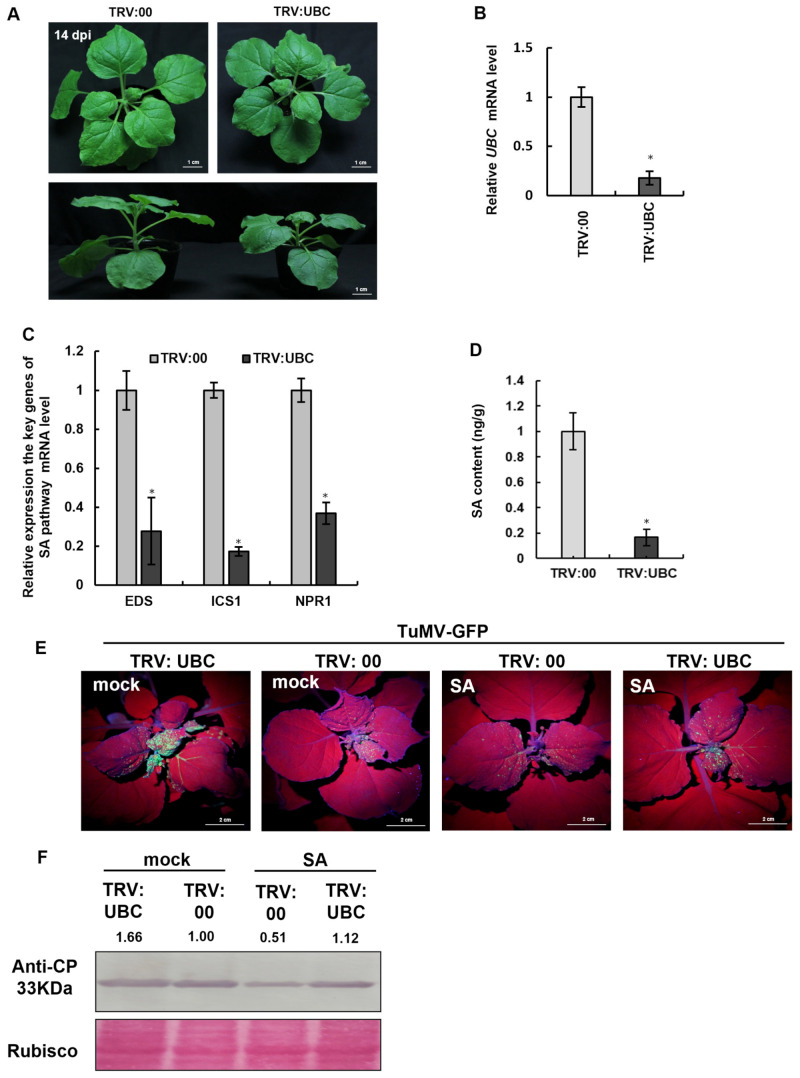
The accumulation of endogenous SA was decreased in UBC-silenced *N. benthamiana*. Decreasing accumulation of the endogenous aalicylic acid (SA) in UBC-silenced *Nicotiana benthamiana*. (**A**) The symptoms in TRV-mediated UBC-silenced *N. benthamiana*. (**B**) After TRV-mediated UBC-silenced *N. benthamiana*, the mRNA level expression of the UBC was examined by RT-qPCR at 14 dpi. Experiments were repeated three times. Bars represent the standard errors of the means; significance was determined by a two-tailed *t*-test (* *p* < 0.05). (**C**) After TRV-mediated UBC silencing, the mRNA level expression of the SA pathway was examined by RT-qPCR at 14 dpi. Experiments were repeated three times. Bars represent the standard errors of the means; significance was determined by a two-tailed *t*-test (* *p* < 0.05). (**D**) The accumulation of endogenous SA was examined in TRV:00 and TRV:UBC-treated WT plants as measured by LC-MS at 14 dpi. The endogenous SA in the TRV:00-treated plant was set as the baseline. Experiments were repeated three times. Bars represent the standard errors of the means; significance was determined by a two-tailed *t*-test (* *p* < 0.05). (**E**) Effect of SA treatment on TuMV-GFP infection in plants inoculated with TRV:00 or TRV:UBC at 7 dpi under UV. Fluorescence intensity differences between independent experiments were attributed to variable Agrobacterium infiltration efficiency, but the trend was consistent across replicates. (**F**) TuMV CP accumulation after SA analog (BTH) treatment. Rubisco served as a loading control; band intensities were quantified with ImageJ and normalized to Rubisco. Although the Rubisco loading band appears lighter in this replicate, the CP signals were quantified after normalization to the corresponding Rubisco signal.

After UBC was subjected to Tobacco rattle virus (TRV)-mediated *UBC* gene silencing, the expression of the UBC gene was detected by RT-qPCR at 14 days post-inoculation (dpi) (post-TRV inoculation). The results showed that UBC gene expression was downregulated (Figure 2D and Figure 4B), and the plants with silenced UBC exhibited stunted growth symptoms (Figure 4A). TuMV-GFP was then infiltrated into UBC-silenced *N. benthamiana* plants using Agrobacterium-mediated infiltration, and at 7 days post-TuMV-GFP inoculation (7 dpi post-TuMV-GFP), viral symptoms were observed under UV light. A notable increase in green fluorescence intensity was observed in UBC-silenced plants (TRV:UBC) versus the control (TRV:00) (Figure 2A). To confirm that silencing of the UBC gene promotes viral infection, Western blot analysis was performed with the TuMV CP antibody. A stronger CP band was detected in TRV:UBC plants, whereas the CP band was weaker in TRV:00, confirming that silencing the UBC gene enhances TuMV-GFP infection (Figure 2B). To further verify that UBC gene silencing promotes TuMV-GFP infection, RT-qPCR detected TuMV CP mRNA expression levels. The results showed that TuMV CP mRNA was significantly higher in TRV:UBC plants compared to TRV:00, further demonstrating that silencing the UBC gene promotes TuMV-GFP infection (Figure 2C).

### 2.3. UBC Overexpression Inhibits TuMV-GFP Accumulation in N. benthamiana

**Figure 3:**
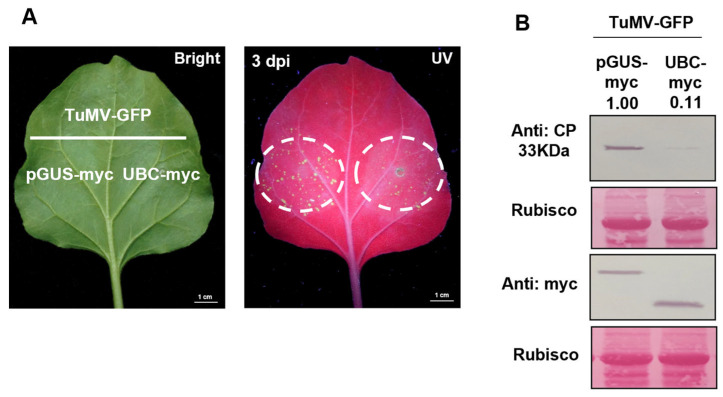
UBC overexpression inhibits TuMV-GFP accumulation in *N. benthamiana*. The accumulation of TuMV-GFP decreased in UBC-silenced *Nicotiana benthamiana*. (**A**) *N. benthamiana* leaves agroinfiltrated with TuMV-GFP and 35S-UBC-myc or 35S-pGUS-myc at 3 dpi. (**B**) TuMV CP accumulation in leaves is indicated in panel (**A**) as revealed by the Western blot analysis with the antibodies against TuMV CP. Myc accumulation was detected with anti-myc antibodies. Rubisco served as a loading control; band intensities were quantified with ImageJ and normalized to Rubisco.

Silencing of UBC enhanced TuMV-GFP accumulation, suggesting that UBC negatively regulates viral infection (Figure 2). Based on this, we hypothesized whether the expression of the UBC protein might promote TuMV-GFP infection. To test this hypothesis, Agrobacterium-mediated transient expression of pGUS-myc, UBC-myc, and TuMV-GFP on *N. benthamiana* was used. At 3 days post-inoculation (dpi) (post-TuMV-GFP inoculation), green fluorescence indicating TuMV-GFP infection was observed under UV light. The results showed that the green fluorescence in plants expressing UBC-myc was weaker compared to that in the control group transiently expressing pGUS-myc (Figure 3A). To further confirm that transient expression of UBC suppresses TuMV-GFP infection, Western blot analysis was used to detect TuMV CP expression. The results revealed that the CP band in the UBC-myc expression was weaker than that in the control group, suggesting that TuMV-GFP infection was inhibited during the transient expression of UBC in *N. benthamiana* (Figure 3B).

### 2.4. The Accumulation of Endogenous SA Decreased in UBC-Silenced N. benthamiana

To investigate the mechanism by which UBC gene silencing inhibits TuMV-GFP infection, we hypothesized that UBC expression may regulate the SA hormonal pathway, and Agrobacterium-mediated TRV-based gene silencing of UBC was performed. To further determine whether the UBC gene expression regulates the gene expression of the SA pathway, RT-qPCR was performed using Nbactin as the internal reference gene to normalize UBC mRNA levels of the key SA pathway genes. The results revealed that the expression levels of EDS1, ICS1, and NPR1 in the SA pathway were markedly downregulated. Furthermore, LC-MS analysis showed a significant decrease in SA accumulation in UBC-silenced *N. benthamiana* plants. These findings indicate that the UBC gene is involved in the biosynthesis of SA (Figure 4C,D).

To further confirm that the UBC regulates SA biosynthesis, TRV-mediated UBC gene silencing (TRV:UBC) was performed. At 14 dpi (post-TuMV-GFP inoculation), the plants were infiltrated with Agrobacterium by TuMV-GFP. Some plants were also treated with the SA analog BHT (benzothiadiazole, 300 µM). At 7 dpi (post-TRV inoculation), green fluorescence was observed under UV and viral accumulation was assessed. The results showed that the SA analog-treated group (TRV:UBC) exhibited less green fluorescence compared to the untreated control group (TRV:UBC), indicating that the SA analog restored SA-mediated disease resistance. Western blot analysis was conducted to detect CP accumulation using the TuMV CP antibody. The results showed that the CP band was weaker in the SA analog-treated group (TRV:UBC) than in the untreated control group (TRV:UBC). Taken together, these results demonstrate that UBC gene silencing suppresses SA accumulation and promotes viral infection (Figure 4D, F).

### 2.5. The Accumulation of Phytohormone JA Was Decreased in UBC-Silenced N. benthamiana

**Figure 5:**
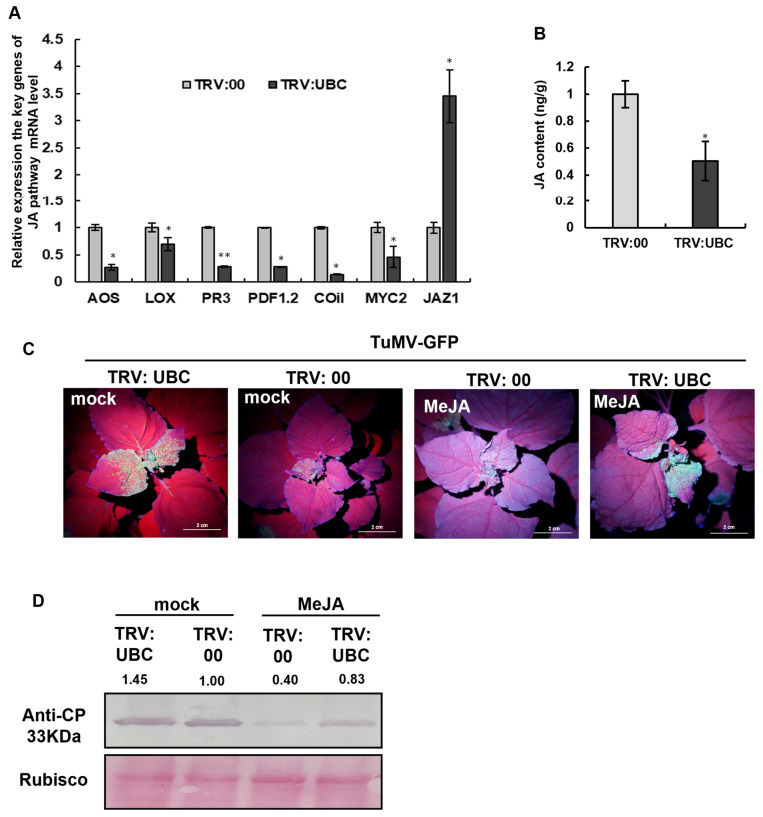
The accumulation of phytohormone JA was decreased in UBC-silenced *N. benthamiana*. Jasmonic acid (JA) accumulates in UBC-silenced *Nicotiana benthamiana*. (**A**) The mRNA level expression of the JA pathway was examined by RT-qPCR at 14 dpi in TRV:00 and TRV:UBC plants. Experiments were repeated three times. Bars represent the standard errors of the means; significance was determined by a two-tailed *t*-test (** *p* < 0.01, * *p* < 0.05). (**B**) The accumulation of endogenous JA was examined in TRV:00 and TRV:UBC-treated WT plants as measured by LC-MS at 14 dpi. The endogenous JA in the TRV:00-treated plant was set as the baseline. Experiments were repeated three times. Bars represent the standard errors of the means; significance was determined by a two-tailed *t*-test (* *p* < 0.05). (**C**) TuMV-GFP infection (7 dpi) in plants inoculated with TRV:00 or TRV:UBC and treated with MeJA. (**D**) TuMV CP accumulation after MeJA treatment. Rubisco served as a loading control; band intensities were quantified with ImageJ and normalized to Rubisco.

To verify whether UBC gene silencing inhibits jasmonic acid (JA) accumulation, Agrobacterium-mediated infiltration of TRV:UBC and TRV:00 was performed on *N. benthamiana* leaves. At 14 days post-inoculation (dpi) (post-TRV inoculation), RT-qPCR was conducted to analyze the expression levels of key genes in the JA signaling pathway. The results showed that the mRNA expression levels of AOS, LOX, PDF1.2, COI1, and MYC2 were significantly downregulated, while the expression level of JAZ1 was markedly upregulated in the TRV:UBC plants. These findings indicate that silencing the UBC gene interferes with the function of JA (Figure 5A).

Furthermore, LC-MS detection showed that the accumulation of the JA hormone was significantly reduced in UBC-silenced *N. benthamiana* plants, confirming that silencing the UBC gene suppresses the function of JA (Figure 5B).

To further elucidate the impact of silencing the UBC gene on JA function, Agrobacterium-mediated infiltration of TRV:UBC and TRV:00 was performed on *N. benthamiana* plants. At 14 dpi, the plants were treated with methyl jasmonate (MeJA), and viral infection was assessed by observing green fluorescence under UV light; virus accumulation was detected by Western blot. The results showed that the MeJA-treated group (TRV:UBC) exhibited less green fluorescence compared to the untreated control group (TRV:UBC), indicating that MeJA application restored JA-mediated resistance against TuMV. Western blot analysis was performed to detect CP accumulation. The results showed that the CP band was weaker in the MeJA-treated group (TRV:UBC) than the untreated control group (TRV:UBC), further supporting the conclusion that UBC silencing inhibits JA accumulation, thereby promoting viral infection (Figure 5C,D).

### 2.6. The Accumulation of RSV and PMMoV Was Increased in UBC-Silenced Plants

**Figure 6:**
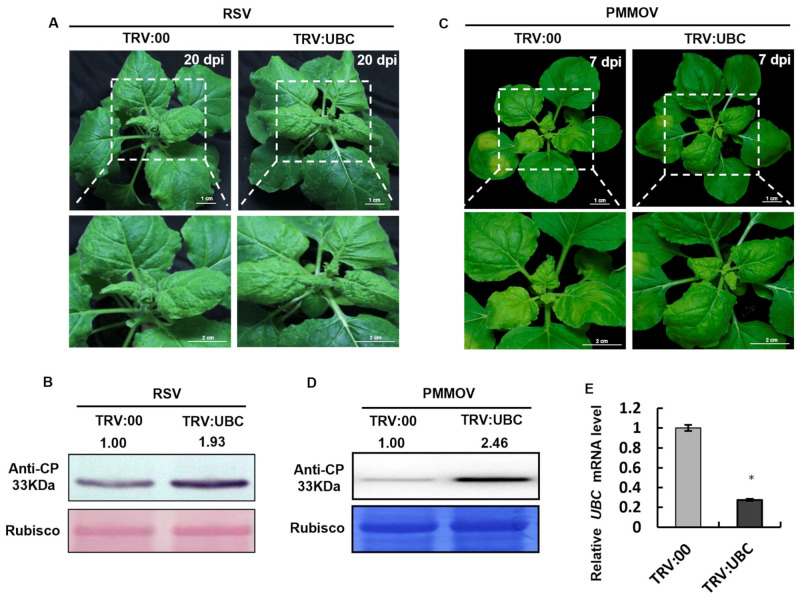
The accumulation of RSV and PMMoV was increased in UBC-silenced plants. The symptoms of RSV and PMMoV were compromised in UBC-silenced *Nicotiana benthamiana*. (**A**) The virus symptoms of RSV infection in TRV:00 and TRV:UBC plants. (**B**) RSV CP accumulation at 20 dpi in TRV:00 and TRV:UBC plants. Rubisco served as a loading control; band intensities were quantified with ImageJ and normalized to Rubisco. (**C**) Effect of PMMoV infection in plants inoculated with TRV:00 and TRV:UBC plants. (**D**) PMMoV CP accumulation in TRV:00 and TRV:UBC plants. Rubisco served as a loading control; band intensities were quantified with ImageJ and normalized to Rubisco. (**E**) After TRV-mediated UBC silencing, the accumulation of UBC was examined by RT-qPCR at 14 dpi. Experiments were repeated three times. Bars represent the standard errors of the means. A two-sample unequal variance directional *t*-test was used to test the significance of the difference (* *p* < 0.05).

To verify whether UBC gene silencing has a general effect on different virus infections, Agrobacterium-mediated infiltration of TRV:UBC and TRV:00 was performed on *N. benthamiana* leaves at 14 days post-infiltration (dpi) (post-TRV inoculation). At 20 dpi (post-RSV inoculation), viral symptoms and accumulation levels were evaluated. The results showed that the TRV:UBC-treated group exhibited more severe symptoms, including significant leaf curling and mosaic patterns, compared to the control group (TRV:00). Western blot analysis revealed that the RSV CP band was stronger in the TRV:UBC group than in the TRV:00 group, indicating that UBC gene silencing enhances RSV infection (Figure 6A,B). In parallel experiments, TRV:UBC- and TRV:00-mediated *N. benthamiana* plants were inoculated with Pepper mild mottle virus (PMMoV). At 7 dpi (post PMMoV inoculation), plant phenotypes were observed visually, and viral accumulation was examined by Western blot. The results showed more severe symptoms in the TRV:UBC, including pronounced leaf wrinkling and chlorosis, compared to the control group. Western blot analysis confirmed higher levels of PMMoV CP accumulation in TRV:UBC-inoculated plants compared to the control group. These findings indicate that UBC gene silencing promotes PMMoV infection (Figure 6C, D). Taken together, these results demonstrate that silencing of the UBC gene enhances susceptibility to multiple viruses, suggesting a broad-spectrum role for UBC in antiviral defense.

## 3. Discussion

Although E3 ubiquitin ligases are known to regulate plant immunity and defend against pathogen infection, studies on the involvement of UBC in plant hormone regulation remain limited [24]. In this study, we found that TuMV-GFP infection significantly increased UBC mRNA accumulation in *N. benthamiana* by RT-qPCR detection, indicating that UBC actively responds during viral infection (Figure 1C). UBC-silenced plants exhibited a stunted phenotype (Figure 4A), and the TuMV-GFP accumulation was markedly enhanced (Figure 2A,B). We hypothesize that the silencing of UBC interferes with immune signaling pathways in plants, thereby promoting viral infection.

Plant hormones play crucial roles in plant defense against viral infection [4]. Viral infection not only induces jasmonic acid (JA)-mediated expression of defense-related genes such as PR3, PR4, and PDF1.2 but also activates other immune pathways, including RNA silencing and brassinosteroid (BR) and salicylic acid (SA) responses [26,27,28]. To successfully infect plants, viruses often interfere with JA biosynthesis or a signaling pathway, particularly the JAZ-MYC regulatory module [29]. Many viruses inhibit JA biosynthesis to weaken plant immunity for promoting viral infection. For example, Rice black-streaked dwarf virus (RBSDV) infection induces the expression of miR319, which targets TCP21 to inhibit JA biosynthesis, suppressing JA-mediated immune responses and facilitating viral infection [30]. The C2 protein of Tomato yellow leaf curl Sardinia virus (TYLCSV) interacts with CNS5 (a component of the COP9 signalosome) to disrupt the interaction between CUL1 and SCF ubiquitination activity, thereby suppressing JA biosynthesis for promoting viral infection [31]. However, how TuMV regulates JA signaling remains unclear. Our results showed that UBC-silenced plants significantly downregulated the expression of key JA biosynthetic and responsive genes, including AOS, LOX, PR3, PDF1.3, and MYC, leading to reduced JA accumulation (Figure 5A).

Deficiencies in nuclear lamina proteins CROWDED NUCLEI (CRWN) induce plant dwarfing and spontaneous cell death lesions, which are caused by the over-production of SA in mutants [32]. We speculated that the stunting phenotype observed in UBC-silenced plants might be associated with the change in SA accumulation. LC-MS analysis confirmed that SA levels were significantly reduced in UBC-silenced plants compared to controls (Figure 4D). Previous studies have reported that endogenous gene mutations in plants can directly or indirectly regulate the SA pathway, affecting both SA accumulation and the expression of related genes [33]. Whether UBC regulates plant hormone biosynthesis and signaling directly or indirectly remains to be elucidated.

The mechanism of UBC-regulated plant hormone-mediated antiviral responses during TuMV-GFP infection was still unclear. SA accumulation was increased by the pub4 mutation of Arabidopsis. PUB4 is a unique E3 ubiquitin ligase; it interacts with the receptor-like kinase CERK1 and negatively regulates the SA pathway [34]. Although previous studies have demonstrated the role of E3 ubiquitin ligases in hormone regulation, our findings show that UBC-silencing leads to significant reductions in JA and SA accumulation, as well as in the expression of related genes, while simultaneously enhancing viral replication. This suggests a close association between UBC and hormone signaling pathways. Further research is needed to fully uncover the underlying mechanisms.

## 4. Materials and Methods

### 4.1. Plant Materials and Agrobacterium Infiltration

Wild-type *N. benthamiana* were grown in pots in a growth room at 24 °C and 60% relative humidity under a 16 h light/8 h dark cycle. The Agrobacterium strain GV3101 was used, and infiltration was performed as described [35]. Equal volumes of individual Agrobacterium cultures (OD600 = 0.5) were mixed before infiltration. GFP fluorescence was observed under UV light (Black Ray model B 100A, Ultra-Violet Products Ltd., Upland, CA, USA) and photographed by a Canon digital camera (EOS 80d). Each treatment group consisted of 12 plants, and experiments were repeated three times.

### 4.2. Virus-Mediated Gene Silencing (VIGS) and Plasmid Constructs

The coding sequence of the UBC gene (GenBank: KR296788.1) was used as the reference to design the PCR primers listed in Table S1. A conserved 295-base pair fragment, which corresponds to the catalytic domain, was targeted for VIGS to ensure specific silencing. This fragment was amplified by PCR from *N. benthamiana* leaf cDNA using Prime STAR HS DNA Polymerase (Takara) under the following cycling conditions: 35 cycles of 98 °C for 30 s, 55 °C for 30 s, and 72 °C for 30 s. The resulting PCR product was purified via gel extraction and subsequently digested with the restriction enzyme PstI (Thermo Fisher Scientific, Massachusetts, USA ). The digested fragment was then ligated into the linearized pTRV2 vector [36] (a gift from Dr. Yule Liu, Tsinghua University) using T4 DNA Ligase. The ligation mixture was transformed into *E. coli* DH5α competent cells, and positive clones were selected and verified by Sanger sequencing, yielding the final construct designated as pTRV2-UBC.

For VIGS, the pTRV1 and pTRV2-derived vectors were transformed into *A. tumefaciens* GV3101. Bacterial cultures carrying pTRV1 and a pTRV2 construct (TRV:00 or TRV:UBC) were mixed at a 1:1 ratio in infiltration buffer (10 mM MgCl_2_, 10 mM MES, pH 5.6, 100 μM acetosyringone). Following a 2–4 h incubation at room temperature, the mixtures were infiltrated into 5-leaf-stage *N. benthamiana* plants. Silencing phenotypes were observed in systemic leaves at 15 days post-infiltration (dpi).

### 4.3. RNA Extraction and RT-qPCR

We extracted total RNA from *N. benthamiana* leaves using TRIzol reagent and treated the RNA with RNase-free DNase I (TransGen, Beijing, China) to remove potential DNA contamination. We performed first-strand cDNA synthesis with oligo(dT)12-18 primers using a Titanium One-Step RT-qPCR Kit (TaKaRa, Tokyo, Japan). For quantitative RT-qPCR, a LightCycler 480 Real-Time PCR System was used for the reaction, and the results were analyzed by the ΔΔCt method. Gene expression was normalized to the internal reference gene Nbactin using the ΔΔCt method. Primers used for RT-qPCR of silencing pathway-related genes and JA or SA-related genes are listed in Table S1.

### 4.4. Plant Hormone Treatment

MeJA was dissolved in 100% ethanol to 50 mM and BTH to 30 mM. Working solutions (50 μM MeJA, 300 μBTH) were prepared by diluting 1:1000 in distilled water. Control plants received 0.1% ethanol only.

### 4.5. Virus Inoculation

The TuMV-GFP genome is depicted from 5′ to 3′. GFP is inserted between P1 and HC-Pro cistrons and flanked by NIa protease cleavage sites (ENLYFQ/G), allowing for its release from the polyprotein upon translation. Downstream elements include P3, 6K1, CI, 6K2, NIa, NIb, and CP (Figure S1). The TuMV-GFP and PMMoV constructs were artificially generated infectious clones preserved in our laboratory. TuMV-GFP and PMMoV were inoculated onto the third true leaf of 4-week-old *N. benthamiana* plants using Agrobacterium strain GV3101 (OD600 = 0.5) via needleless syringe infiltration. Agrobacterium-mediated infiltration of Rice stripe virus (RSV) was performed on the plants as described previously [35,37,38].

### 4.6. Protein Analysis

Total proteins for the Western blot (WB) assay were extracted from leaf tissues as described [35,38]. The following proteins were detected by specific antibodies: anti-TuMV CP (produced by the authors’ lab), anti-PMMoV CP (produced by the author’s lab), anti-RSV CP (produced by the authors’ lab), and alkaline phosphatase-conjugated goat anti-rabbit IgG (TransGen, Beijing, China)) served as a secondary antibody (1:10,000). Commercial mouse antibodies: anti-myc (TransGen, HT101-01). Alkaline phosphatase-conjugated goat anti-mouse IgG (TransGen) served as a secondary antibody (1:10,000). The WB was visualized using nitrotetrazolium blue chloride/5-bromo-4-chloro-3-indolyl phosphate (NBT/BCIP) buffer (Sigma-Aldrich, St. Louis, USA).

### 4.7. Detection of Hormone Content

*N*. *benthamiana* leaf tissues (~900 mg) were analyzed by high-performance liquid chromatography–tandem mass spectrometry (LC-MS) with JA-type and SA-type samples (Sigma-Aldrich) according to a method previously described [39]. Three independent replicates, each containing three biological repeats, were used for hormone quantification. Hormone levels were measured by Nanjing Ruiyuan Biotechnology Co., Ltd., Nanjing, China.

## 5. Conclusions

In conclusion, our study explored the mechanism of the ubiquitin-conjugating enzyme (UBC) during turnip mosaic virus (TuMV-GFP) infection in *Nicotiana benthamiana*. TuMV-GFP infection was promoted in *UBC*-silenced *Nicotiana benthamiana*, and a quantitative analysis of JA and SA in UBC-silenced plants by LC-MS revealed a reduction in the accumulation of salicylic acid (SA) and jasmonic acid (JA) hormones in the leaves. Moreover, silencing the UBC gene also showed broad-spectrum promotion effects on Rice stripe virus (RSV) and Pepper mild mottle virus (PMMoV). This study provides a theoretical basis for understanding the interaction mechanisms between plants and viruses, and it offers important insights for developing broad-spectrum antiviral strategies based on plant hormone signaling pathways.

## Data Availability

The data presented in this article are available upon request from the corresponding author.

## Supplementary Materials

The following supporting information can be downloaded at: https://www.mdpi.com/article/10.3390/plants14203234/s1, Table S1: Oligonucleotides used for RT-qPCR and cloning; Figure S1: TuMV-GFP infectious clone GFP green fluorescence insertion genomic position.
