# Supplementary material for: Ubiquitin-Conjugating Enzyme Positively Regulates Salicylic Acid and Jasmonic Acid Biosynthesis to Confer Broad-Spectrum Antiviral Resistance in Nicotiana benthamiana
Source: Plants (Basel). 2025 Oct 21;14(20):3234. doi: 10.3390/plants14203234 (PMC12567447; doi:10.3390/plants14203234)
Supplement: Supplementary file 1 [file plants-14-03234-s001.zip › plants-3829088-supplementary.pdf]

A

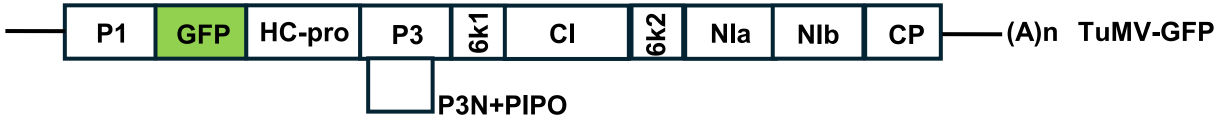

Figure S1. TuMV-GFP infectious clone GFP green fluorescence insertion genomic position.

(A) Schematic representation of the genomes of TuMV with GFP (TuMV-GFP) genes inserted between P1 and HC-Pro cistrons.
